# A long-term survival case with proton beam therapy for advanced sphenoid sinus cancer with hypopituitarism

**DOI:** 10.1007/s13691-021-00524-9

**Published:** 2021-11-27

**Authors:** Yojiro Ishikawa, Motohisa Suzuki, Hisashi Yamaguchi, Ichiro Seto, Masanori Machida, Yoshiaki Takagawa, Keiichi Jingu, Yasuyuki Kikuchi, Masao Murakami

**Affiliations:** 1 Department of Radiation Oncology, Southern Tohoku Proton Therapy Center, 7-172, Yatsuyamada, Koriyama, Fukushima 963-8052 Japan; 2 Department of Radiation Oncology, Tohoku University Graduate School of Medicine, 1-1, Seiryo-chou, Aoba-ku, Sendai, 980-8574 Japan

**Keywords:** Proton beam therapy, Sphenoid sinus cancer, Secondary hypopituitarism, Radiation-induced optic neuropathy

## Abstract

Sphenoid sinus malignancies are rare diseases. Secondary hypopituitarism associated with sphenoid sinus malignancy is not well known. A 41-year-old male complained of right ptosis. Neurological findings revealed right oculomotor, trochlear and glossopharyngeal nerve palsy. Imaging diagnosis suggested a tumor that had spread bilaterally from the sphenoid sinus to the ethmoid sinus, nasopharynx and posterior pharyngeal space. Biopsy revealed squamous cell carcinoma (SCC). Based on these findings, a clinical diagnosis of SCC of the sphenoid sinus was made. Removal of the tumor without damaging nearby organs would have been difficult because the tumor extended to the bilateral optic nerves, optic chiasma and internal carotid artery, and surgeons, therefore, recommended proton beam therapy (PBT). Before PBT, the hypopituitarism occurred in the patient and we administered hydrocortisone and levothyroxine. During treating for hypopituitarism, we performed PBT with nedaplatin and 5-fluorouracil. The daily PBT fractions were 2.2 relative biological effectiveness (RBE) for the tumor received total dose of 81.4 Gy RBE. The acute side effect of grade 2 dermatitis according to the National Cancer Institute Common Terminology Criteria for Adverse Events version 4.0. Occurred after PBT. The patient needs to take hydrocortisone and levothyroxine, but he remains in complete remission 8 years after treatment without surgery or chemotherapy. Visual function is gradually declining, but there is no evidence of severe radiation-induced optic neuropathy.

## Introduction

Sphenoid sinus malignancies are rare diseases, accounting for less than 1% of all malignancies. Although sphenoidal sinus malignancies commonly cause headache, diplopia, and various other cranial neuropathies [1, 2] it is not well known that sphenoid sinus malignancies can occur with secondary hypothyroidism. There are only a few reports on the management of secondary hypopituitarism-indued sphenoid sinus malignancies.

Recently, proton beam therapy (PBT) has been used for malignancies in sphenoid sinus because protons have excellent dose localization according to the Bragg peak compared with photons and are biologically equivalent to conventional X-ray treatment for cancer [3, 4].

We herein report the achievement of a long-term survival case with PBT for advanced sphenoid sinus cancer with hypopituitarism.

## Case report

**Fig. 1:**
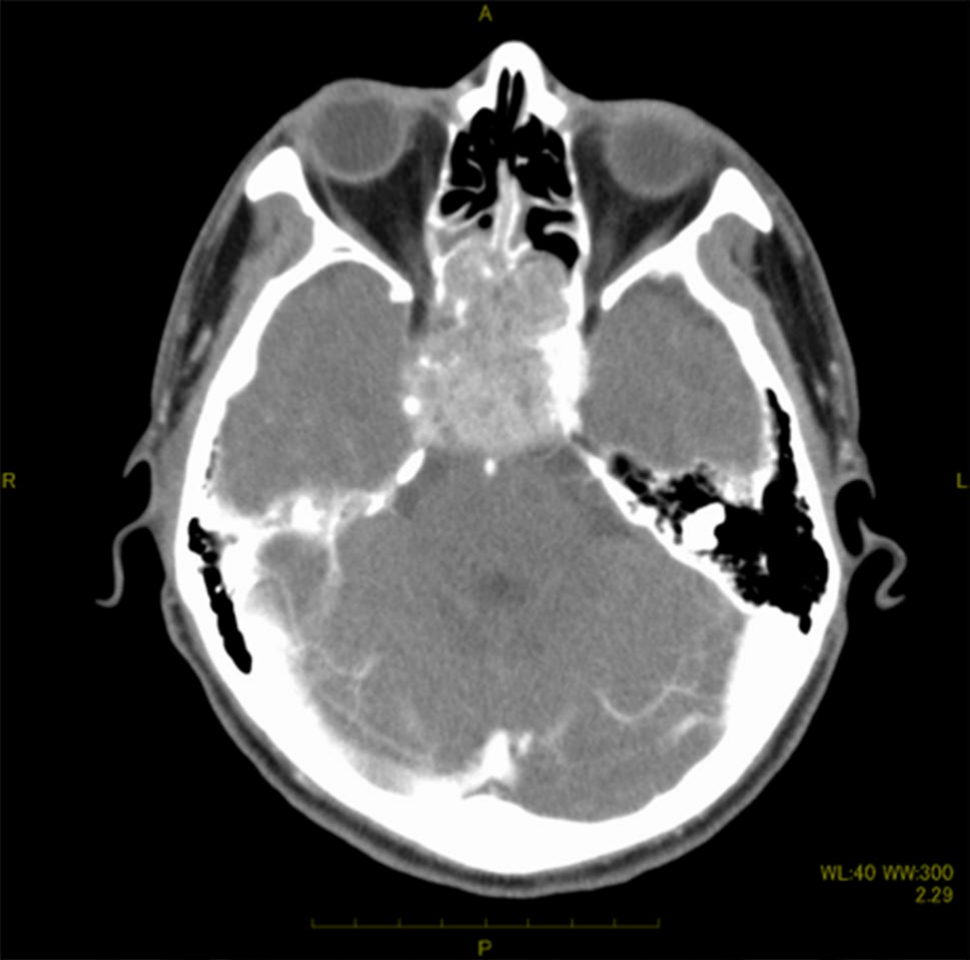
Contrast-enhanced computed tomography scan images revealed a tumor that had spread bilaterally from the sphenoid sinus to the ethmoid sinus and showed multiple honeycomb-like low-density areas and suggested skull base infiltration

**Fig. 2:**
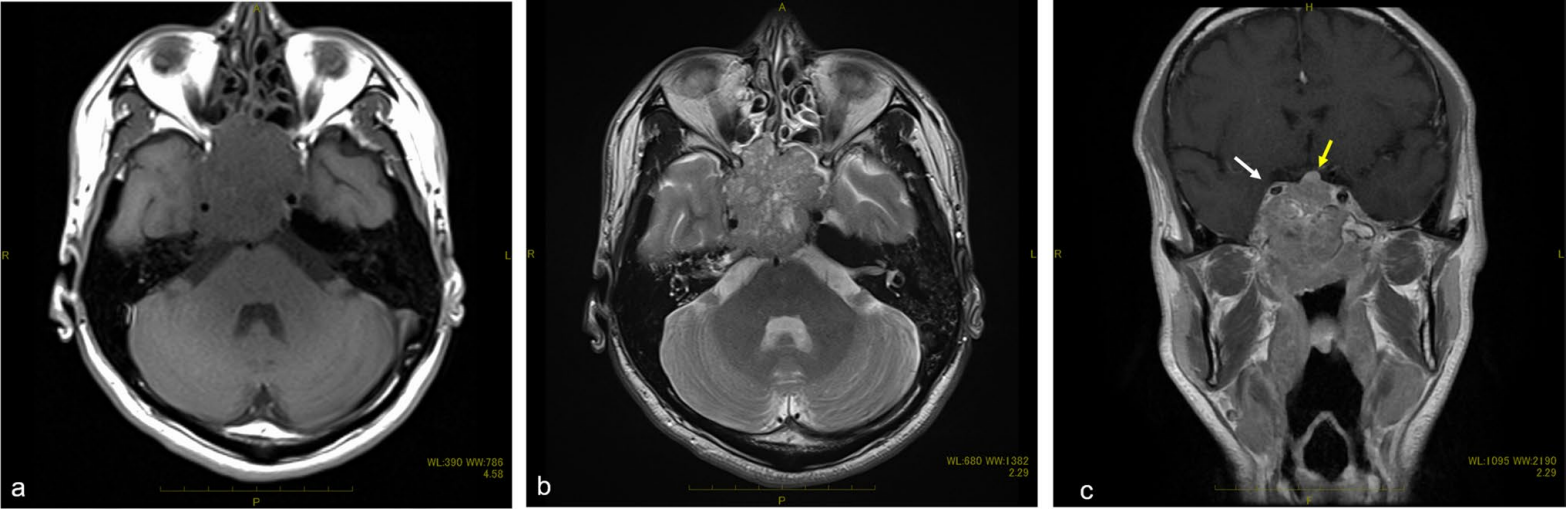
Magnetic resonance imaging: the tumor showed lower and intermediate intensity than the brain parenchyma on a T1W1 (**a**) and intermediate intensity compared to the brain parenchyma and with many high-intensity on a small cysts at T2WI (**b**). On the gadolinium-enhanced T1WI shoed a non-uniform enhancing tumor that compressed the pituitary gland (yellow arrow) and extended to the right spongy pulsation (white arrow) (**c**)

**Fig. 3:**
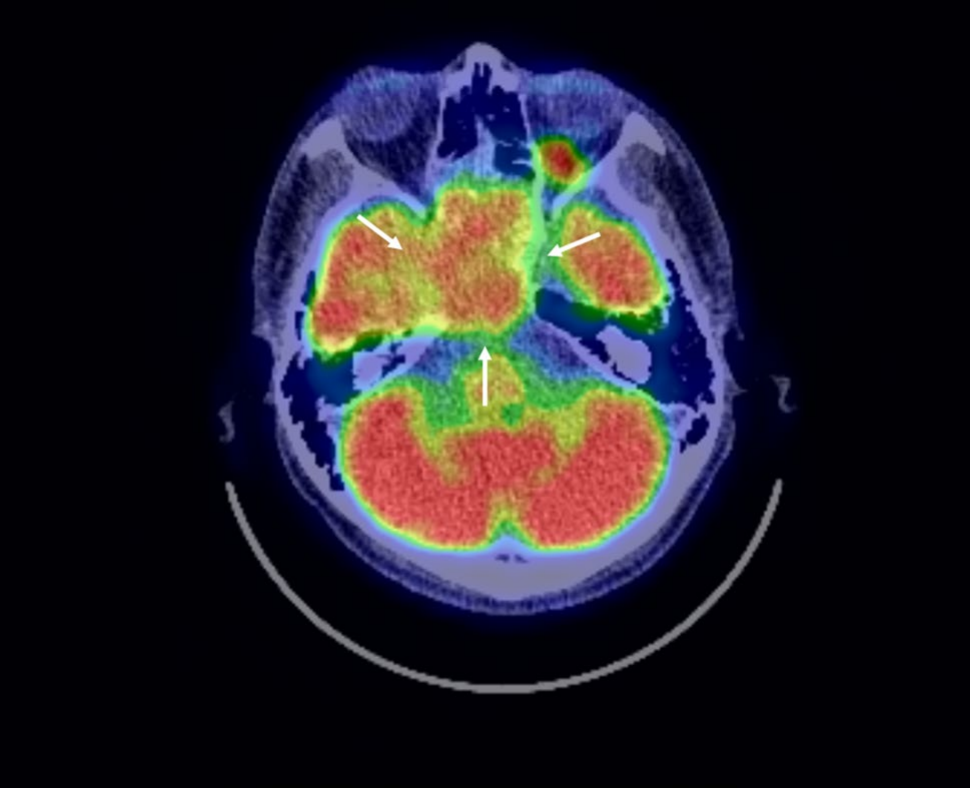
Positron emission tomography-CT (PET-CT) showed uptake of 18F-2-fluoro-2-deoxy-d-glucose in the sphenoid sinus (maximum standardized uptake value of 15.83) (white arrow)

**Fig. 4:**
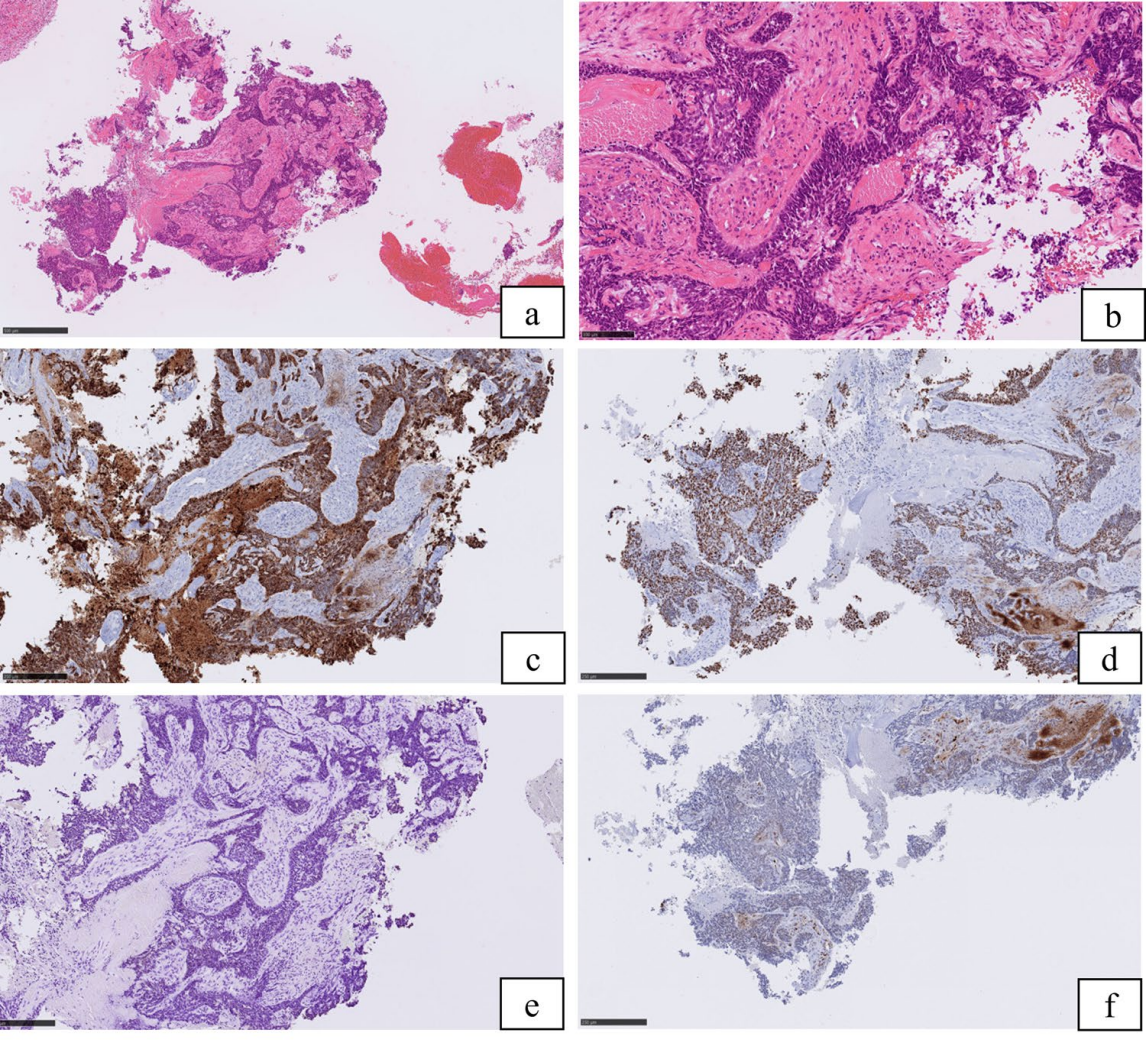
Histopathology showed a squamous cell carcinoma. [hematoxylin and eosin stain; × 5 (**a**) and × 20 (**b**)]. Immunohistochemical staining of the tumor revealed CK5/6-positive (**c**), p40-positive (**d**), Ber-EP4-negative (**e**) and p16-negative (**f**)

A 41-year-old male complained of right ptosis. His medical history was schizophrenia, but he was stable with risperidone. There was no history of drinking or smoking and no exposure to organic solvents, welding fume and arsenic. Neurological findings revealed right oculomotor, trochlear and glossopharyngeal nerve palsy. The corrected visual acuity was 1.2 in right eye and 1.2 in the left. Computed tomography (CT) revealed a tumor which spread bilaterally from the sphenoid sinus to the ethmoid sinus, nasopharynx and posterior pharyngeal space (Fig. 1). MRI revealed an irregular tumor centered on the sphenoid sinus and clivus. Gadolinium-enhanced T1WI showed non-uniform enhancing tumor that had compressed the pituitary gland and extended to the right spongy pulsation (Fig. 2). Positron emission tomography-CT (PET-CT) revealed uptake of 18F-2-fluoro-2-deoxy-D-glucose in the sphenoid sinus (maximum standardized uptake value of 15.83) (Fig. 3). Imaging diagnosis suggested adenocarcinoma, squamous cell carcinoma (SCC), adenoid cystic carcinoma, and metastatic tumor of the sphenoid sinus. Histological examination of biopsy specimens from the nasopharynx was diagnosed as SCC. Immunohistochemical staining of the tumor revealed CK5/6 ( +), p40 ( +), Ber-EP4 ( −) and p16 ( −) (Fig. 4). Based on these findings, the clinical diagnosis before treatment was SCC of the sphenoid sinus. The tumor extended to the bilateral optic nerves, optic chiasm and internal carotid artery, and surgeons, therefore, recommended PBT because it was difficult to perform resection. They introduced the patient to our PBT center for PBT as an alternative treatment. The patient was given an explanation about the risk of radiation-induced optic neuropathy after PBT because of the bilateral optic nerve infiltration of the tumor. He agreed to receive PBT in favor of tumor control rather than vision preservation.

**Fig. 5:**
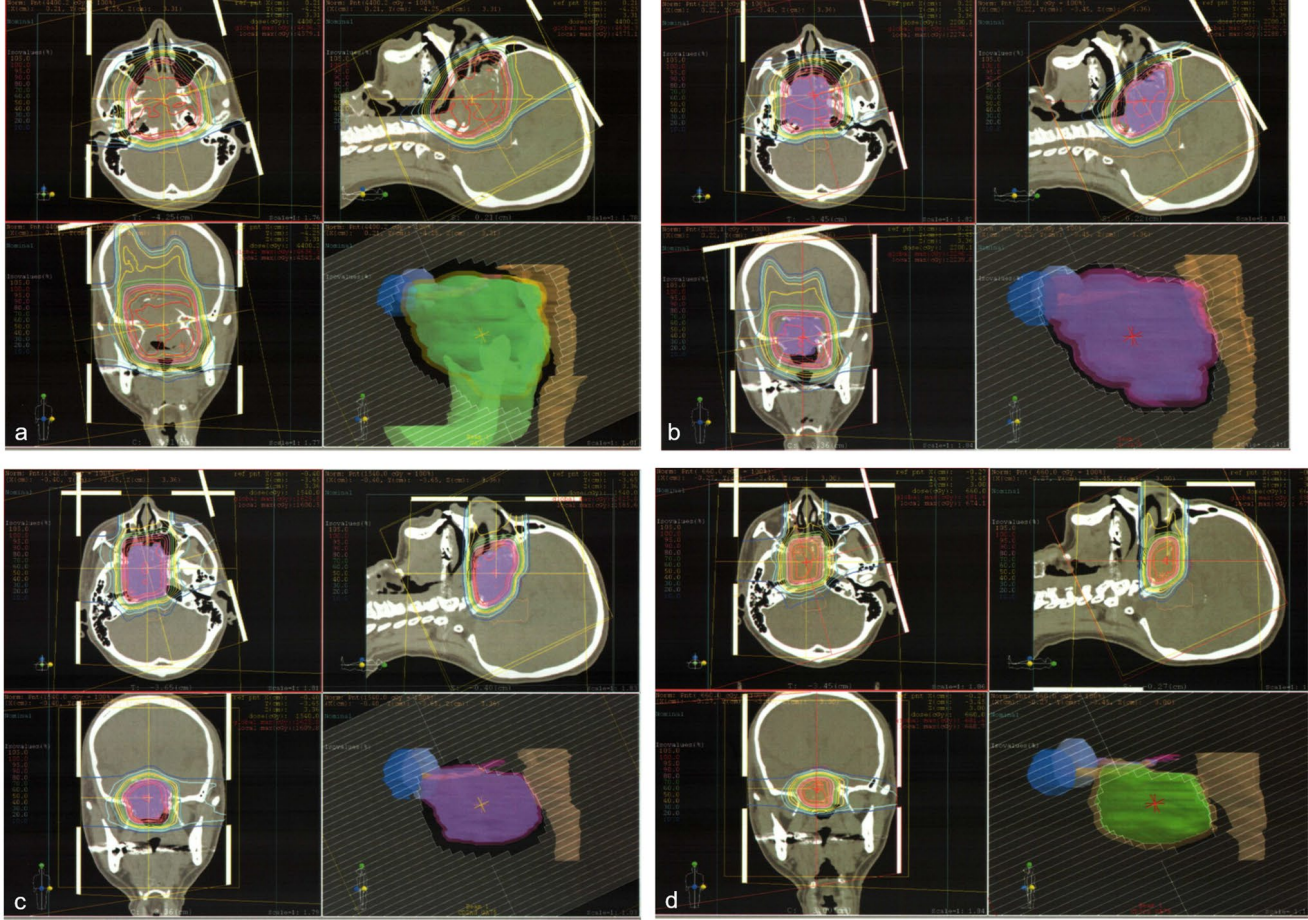
Dose distribution of proton beam therapy. **a** The initial field was treated with 39.6 Gy relative biological effectiveness (RBE) in 18 fractions. **b** The first replan field were treated with 22 Gy RBE in 10 fractions. (cumulative dose of 61.6 Gy RBE). **c** The second replan field was treated with 13.2 Gy RBE in 6 fractions (The cumulative dose of 74.8 Gy RBE). **d** The final field was treated with 6.6 Gy RBE in 3 fractions (The cumulative dose of 81.4 Gy RBE)

Before PBT, he was admitted to our hospital urgently due to general malaise and difficulty in eating food. Laboratory investigations showed a fasting blood glucose level of 78 mg/dL and a serum sodium level of 127 mEq/L. According to laboratory data and a previous imaging examination, we considered the possibility of increased intracranial pressure due to the sphenoid sinus carcinoma. We used concentrated glycerin and dexamethasone as much as possible. Although the general condition of the patient improved, urine volume became 7000 ml per day. Laboratory investigations also revealed a cortisol level of less than 1.00 µg/dL (normal range 4.0–18.3), an ACTH level of less than 2.0 pg/mL (normal range 7.2–63.3), an FT4 level of 0.53 ng/dL (normal range 0.9–1.7), a TSH level of 0.13 mIU/L (normal range 0.50–5.0), a prolactin level of 10.3 µg/L (normal range 4.29–13.69), an FSH level 0.34 mIU/L (normal range 2.0–8.3), and a LH level 0.12 mIU/L (normal range 0.79–15.72). We, therefore, finally diagnosed hypopituitarism and treated the patient with dexamethasone (0.5 mg) and levothyroxine (50 μg). During treating for hypopituitarism, we performed two courses of chemotherapy with PBT. We delivered nedaplatin of 70 mg/m2 because of avoiding fluid loading and a continuous infusion of 5-fluorouracil at 1000 mg/m2 over a 24-h period. The PBT system at our institute (Proton beam system, Mitsubishi, Tokyo, Japan) uses synchrotron and scattering methods. The gross tumor volume (GTV) included the sphenoid sinus tumor. The clinical target volume (CTV) was defined as GTV plus 0.5-cm margins. The planning target volume (PTV) was CTV plus 0.5-cm margins. The daily PBT fractions were 2.2 relative biological effectiveness (RBE) for sphenoid sinus carcinoma that received a total dose of 81.4 Gy RBE with replanning three times (Fig. 5). The maximum doses to the optic nerves and chiasma were obtained from dose–volume histograms (DVHs). The maximal cumulative dose to the right and left optic nerves and optic chiasma were 74.9, 72.6, and 68.5 Gy RBE, respectively.

**Fig. 6:**
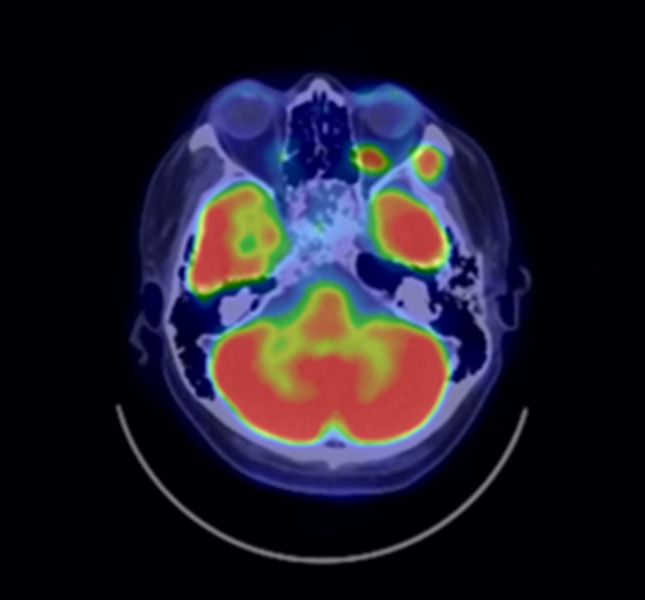
PET-CT after proton beam therapy (PBT). PBT resulted in the disappearance of fluorodeoxyglucose in the sphenoid sinus

**Fig. 7:**
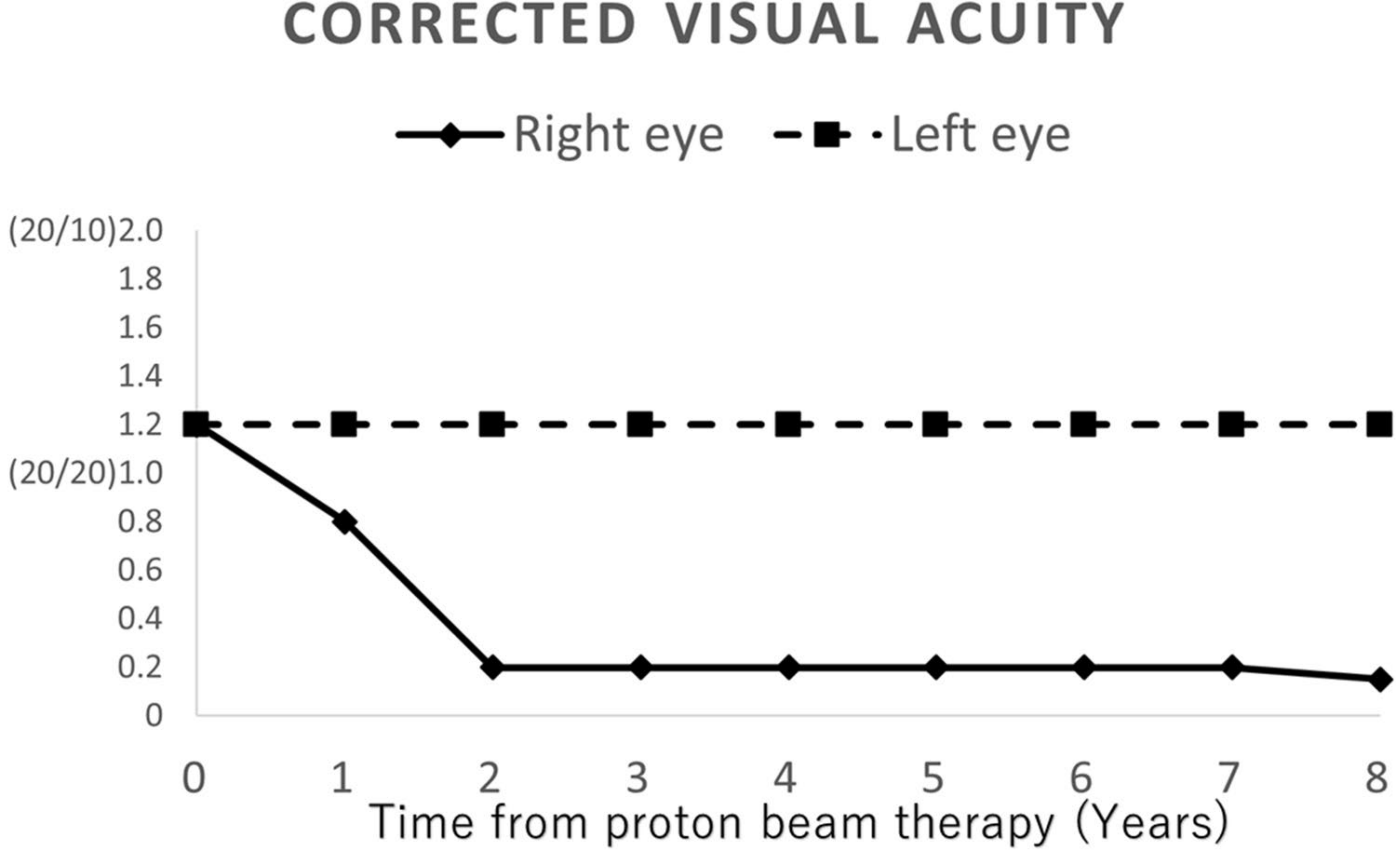
The corrected visual acuity after proton beam therapy for advanced sphenoid sinus cancer

An acute side effect of grade 2 dermatitis according to the National Cancer Institute Common Terminology Criteria for Adverse Events version 4.0. occurred after PBT, but there was no acute complication of more than grade 3. PET after treatment showed no evidence of recurrence (Fig. 6). Laboratory investigations four weeks after PBT revealed a cortisol level of less than 1.00 µg/dL, an FT4 level of 1.27 ng/dL (normal range 0.9–1.7), a TSH level of 0.923 mIU/L (normal range 0.50–5.0), a prolactin level of 7.81 µg/L (normal range 4.29–13.69), an FSH level 10.81 mIU/L (normal range 2.0–8.3), and a LH level 4.66 mIU/L (normal range 0.79–15.72). The patient needs to take triamcinolone acetonide (4 mg) and levothyroxine (50-75 μg), but he remains in complete remission 8 years after treatment without surgery or chemotherapy. The corrected visual acuity is gradually declining in the right eye, but there is no evidence of sever radiation-induced optic neuropathy in the left eye (Fig. 7).

## Discussion

Sinonasal malignancies (SNM) are extremely rare, accounting for only 1% of all malignancies. Sphenoid sinus cancer is an even rarer disease, accounting for about 0.3–4.1% of SNM [1, 5–7]. Sphenoid sinus cancer commonly occurs in males and at an age around 50 years [1, 2]. Among epithelial malignant tumors, SCC is the most common (33–40%) and followed by adenocarcinoma [1, 8]. SNM included sphenoid sinus cancer epidemiologically associated with occupational exposure to wood, leather and textiles, dust, organic solvents, welding fumes, or arsenic [9, 10]. There was no such exposure in our case. HPV-positive head and neck tumors generally have good overall survival [11]; however, immunohistochemical staining of the tumor revealed p16-negative in the present case. Since sphenoid sinus malignancies are close to the base of the skull, they are often different from other sinus malignancies and often have neurological symptoms including cranial neuropathy. The types of tumor progression include orbital, nasal, cranial (cranial nerves I, II, III, IV, V), petrous (cranial nerves VII and VIII) and occipital extensions [1, 2]. Our case was accompanied by an orbital, nasal, cranial, petrous and occipital extensions.

The incidence of hypopituitarism about four per 100,000 per year and the rate of mortality associated with hypopituitarism is high [12]. Secondary hypopituitarism rarely occurs in patients with sphenoid sinus disease [12, 13]. Lebovits et al. reported a patient with sphenoidal sinus carcinoma causing symptomatic panhypopituitarism and they also reported that secondary hypopituitarism occurred in 1% of sphenoid sinus malignancies [13]. Hypopituitary patients exhibit increased incapacitation and sick days, low health status and low quality of life. To the best of our knowledge, there has been no report of treatment for SCC in the sphenoid sinus with hypopituitarism.

The 2-year survival rate for patients with SCC in the sphenoid sinus was reported to be 44% [1]. A total irradiation dose of 50–70 Gy has been used for radiation therapy for head and neck cancer including sphenoid sinus carcinoma with or without chemotherapy [15]. Although conventional X-ray treatment cannot provide a target with more than 70 Gy because of the organ at risk, PBT can provide a sufficiently high dose. Minhet et al. also reported that primary tumors in the sphenoid sinus received PBT at a total dose of 76 Gy RBE and that the 2-year overall survival rate was 53%. They also reported that the 2-year overall survival rate for patients with tumors extending beyond the sphenoid sinus was 19% [16].

In the present case, PBT provided a sufficiently high dose for the sphenoid sinus. However, the bilateral optic nerves were subjected to PBT, and the corrected visual acuity is gradually declining in the right eye. Although PBT is a technology that is designed to further reduce the amount of radiation that affects the surrounding normal tissue than intensity modulated radiation therapy (IMRT), IMRT might have been also sufficient for the treatment in this case. The optic nerve in this patient was included in the irradiation field of PBT; however, carbon ion therapy might be able to spare the optic nerves. Koto et al. reported the use of carbon ion therapy for 458 patients with SNM tumors including 11 patients with sphenoid sinus malignant tumors, and the 2-year overall survival rate was 79.6%. The median follow-up period was 25.2 months (ranging from 1.4 to 132.3 months) [17]. Although the number of patients in each group was small, a trend toward improved survival was noted in patients who received PBT or carbon ion therapy. The present case achieved and remained in complete remission for 8 years after PBT.

Radiation-induced optic neuropathy (RION) is a complication of radiation therapy including PBT. In our case, we could not avoid the bilateral optic nerves and chiasma because the tumor location was close to the bilateral optic nerves and chiasma. Our case, therefore, received more than 60 Gy RBE to the optic nerves and chiasma. The 10-year actuarial risk of RION for patients treated with 61–78 Gy has been reported to be 24–30% [18]. Puyao et al. also reported that the cumulative incidence of RION after PBT was 5.8% in patients receiving more than 60 Gy RBE to the optic pathway [19]. Advanced age and female sex were reported to be associated with a risk of RION [19, 20]. In addition, treatment with steroid has demonstrated some efficacy for RION [21]. Our patient was young male and medication with hydrocortisone or triamcinolone acetonide might have decreased the risk of RION.

Because this study is a case study, it is difficult to define the indication for SCC in sphenoid sinus. However, it is possible that some patients with advanced sphenoid sinus malignancies have been treated with only insufficient resection or radiation therapy despite being potential candidates for PBT. We believe that this case will have a considerable impact on the therapeutic option for SCC in the sphenoid sinus. We hope that the indications of sphenoid sinus malignances will be expanded.
